# Finite Element Analysis on Initial Crack Site of Porous Structure Fabricated by Electron Beam Additive Manufacturing

**DOI:** 10.3390/ma14237467

**Published:** 2021-12-06

**Authors:** Meng-Hsiu Tsai, Chia-Ming Yang, Yu-Xuan Hung, Chao-Yong Jheng, Yen-Ju Chen, Ho-Chung Fu, In-Gann Chen

**Affiliations:** 1 Department of Mold and Die Engineering, National Kaohsiung University of Science and Technology, Kaohsiung 807618, Taiwan; tmh@nkust.edu.tw (M.-H.T.); terry840710@yahoo.com.tw (Y.-X.H.); 2 School of Dentistry, College of Dental Medicine, Kaohsiung Medical University, Kaohsiung 80708, Taiwan; 3 Department of Materials Science and Engineering, National Cheng-Kung University, Tainan 701, Taiwan; lf2killer@hotmail.com (C.-M.Y.); water60068@gmail.com (C.-Y.J.); 4 Metal Processing R & D Department, Metal Industries Research and Development Centre (MIRDC), Kaohsiung 811, Taiwan; yjchen@mail.mirdc.org.tw

**Keywords:** electron beam additive manufacturing, Ti6Al4V, brittleness, finite element analysis, elastic model, initial crack site

## Abstract

Ti6Al4V specimens with porous structures can be fabricated by additive manufacturing to obtain the desired Young’s modulus. Their mechanical strength and deformation behavior can be evaluated using finite element analysis (FEA), with various models and simulation methodologies described in the existing literature. Most studies focused on the evaluation accuracy of the mechanical strength and deformation behavior using complex models. This study presents a simple elastic model for brittle specimens followed by an electron beam additive manufacturing (EBAM) process to predict the initial crack site and threshold of applied stress related to the failure of cubic unit lattice structures. Six cubic lattice specimens with different porosities were fabricated by EBAM, and compression tests were performed and compared to the FEA results. In this study, two different types of deformation behavior were observed in the specimens with low and high porosities. The adopted elastic model and the threshold of applied stress calculated via FEA showed good capabilities for predicting the initial crack sites of these specimens. The methodology presented in this study should provide a simple yet accurate method to predict the fracture initiation of porous structure parts.

## Highlights

1. A combination of EBAM and FEA was employed for the analysis of initial crack sites.
2. The initial crack sites predicted by the elastic model were consistent with experimental results.
3. The threshold stresses calculated by FEA coincided with the crack sites of specimens with different porosities.
4. The collapse mechanism due to strut behavior under uniaxial compression stress was investigated.

## 1. Introduction

Porous materials, also called cellular solids, fabricated by additive manufacturing have attracted attention in biomedical implants, such as acetabular hip cups [1,2,3], mandibles [4], fusion cages [5,6], and various other applications [2]. Implants made from a material with a low elastic modulus can reduce the effect of stress shielding [7]. The use of porous metal structures can effectively eliminate this phenomenon. The amount of porosity in the implant is considered a crucial factor in promoting successful bone integration with a porous structure [8]. Conventional methods of fabricating porous materials include chemical and heat treatment [9], powder metallurgy [10], and thermal spraying [11]. However, these manufacturing techniques have limitations in terms of controlling the size, shape, and distribution of the pores. A state-of-the-art method for fabricating porous materials is powder bed fusion (PBF), which is a process of fusing powder materials to make objects according to a 3D model, usually by layer-upon-layer melting with a laser or electron beam heating source. Selective laser melting (SLM) and electron beam additive manufacturing (EBAM), as a kind of metal powder bed layer-by-layer fusion process, provide considerable freedom to designers for deciding the geometry of the final part and allow for a reduced manufacturing time.

SLM/EBAM-fabricated parts present porous structures with variations in topological designs and porosities (i.e., the volume ratio of the porous structure and bulk) to obtain the desired Young’s modulus. Gibson and Ashby [12] reported an exponential square formula for the relationship between the relative density and mechanical properties of porous structures, such as Ti6Al4V cubic-type porous structures with porosities ranging from 60% to 96.2%, with the final effective elastic modulus ranging from 23 MPa to 78 MPa [13]. For approximately the same porosities of 49.75% and 50.75%, with variations only in the strut thicknesses of specimens, their compressive stiffness and strength values decreased significantly from 2.92 GPa to 0.57 GPa (80.5% reduction) and from 163.02 MPa to 7.28 MPa (93.54% reduction), respectively [14]. Li et al. investigated a Ti6Al4V porous structure with different meshes (cubic, G7, and rhombic dodecahedron) fabricated by the EBAM process [15]. In that research, the influence of porosity was the main discussion point by observing specimens’ compressive mechanical properties at each density, However, the influence of porosity gradients was not discussed. Grunsven et al. studied a Ti6Al4V diamond-type lattice structure fabricated by EBAM with variations in the porosity [16]. The collapse of the porous gradient structure of specimens with cubic and honeycomb lattices started from a less dense layer and progressed to more dense layers, as reported by Choy et al. [17]. Most of these studies showed a power-law relationship between the porosity and elastic modulus, similar to that given by Gibson and Ashby’s equation. In addition, the plastic deformation behavior revealed the coupling of buckling and bending in the compression stress–strain curve.

Finite element analysis (FEA) is a technique commonly used to evaluate the mechanical behavior of porous materials. By using FEA, evaluations of overall and local stress distribution in a unit cell structure could be shown for further research purposes. Therefore, FEA-based simulations were used to estimate the mechanical properties and determine the failure modes of the lattice structures. For elastic modulus studies, some researchers reported the elastic modulus discrepancies between computational and experimental results [18,19]. The elastic modulus predicted with finite element modeling was as much as 10 times higher than that obtained via experiments with a Ti6Al4V simple cubic porous structure fabricated by EBAM [19]. It was proposed that only perfect elastic behavior, rather than elastic–plastic behavior, contributes to enlarging the difference between the theoretical and experimental results. The porosity of the porous structure can be changed by altering the strut or pore size. It has been reported that cell topologies, strut diameters, cell sizes, bulk geometries, and strut defects affect the mechanical properties owing to local stress concentrations [20,21,22,23]. As for the deformation of deformation behavior in simulation, Ushijima et al. [24] reported an FEA to predict the initial stiffness and plastic collapse strength of body-centered cubic (BCC) lattice structures under compressive loads. The analytical and FEA predictions were compared with the experimental results. A good agreement was found between the experimental data and the corresponding FEA predictions for structures with a low relative density. The collapse modes for BCC and BCC with vertical pillars were evaluated in the FEA models, and they were also in agreement with the experimental observations [25].

Most of these numerical simulations adopted complex nonlinear models to predict deformation behavior and compared the obtained results with microcomputed tomography (Micro-CT) images to prove the accuracy of evaluation, as well as determine the elastic modulus, collapse strength, and macroscale deformation behavior. There is limited research on the failure behavior of the initial site as a function of the stress distribution of porous titanium parts fabricated by EBAM process. Therefore, it is important to illustrate the stress distribution behavior to gain an understanding of the local stress concentration and fracture mechanism. Additionally, EBAM-fabricated porous specimens with martensitic microstructures that exhibit brittle properties have been previously reported by us [5] and other researchers [14,15]. This study aimed to predict the failure at initial sites and the threshold stress causing the failure of high-porosity specimens using a simple elastic model, which differs from the elastic–plastic FEAs in the literature reporting on porous Ti6Al4V compressive specimens. Thus, we present cubic porous titanium parts fabricated by EBAM technology, with different porosities ranging from 25% to 63%. Compression tests were performed on these EBAM-fabricated specimens to study the failure behavior, and in situ digital images were acquired. In addition to the aforementioned failure at the initial points, FEA results of the cubic-type porous structure were analyzed with regard to the local stress concentration for comparison with the experimental observations. The objective of this study was to evaluate the crack initiation sites of compressed Ti6Al4V porous parts fabricated by EBM, as well as the threshold stresses of porous specimens at fracture.

## 2. Materials and Methods

### 2.1. Specimen Preparation

**Figure 1:**
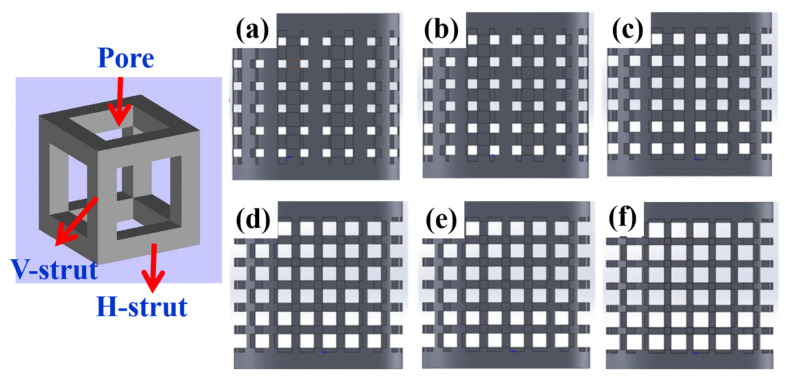
Schematic diagram for cubic unit cell pore and strut morphologies with side view of the designed Ti6Al4V porous structures from (**a**–**f**). Long axis of the vertical struts denotes that the V-strut was parallel to the compressive force direction, while the horizontal ones denote that the H-strut was vertical to the direction of the force. Specimens with different porosities based on the strut size change from 25% to 63% in porosity are shown: (**a**) 25%, (**b**) 33%, (**c**) 40%, (**d**) 55%, (**e**) 59%, and (**f**) 63%.

To investigate the porosity and mechanical properties of EBAM-fabricated porous metallic parts, several specimens according to ASTM-E9 89a standards were designed by Inventor (2017) and manufactured by the EBAM process. The specimens were cubic-type porous structures with fixed cell sizes of 4 mm, nominal strut diameters of 0.6 to 1.25 mm, and pore diameters of 1.5 to 2.8 mm, making a total of six different porosity conditions of the specimens in Table 1; unit cell models are shown in Figure 1.

### 2.2. EBAM Process

In this study, specimens with different porosities were fabricated on an Arcam Q10 machine (Arcam EBM GE Additive Company, Sweden), the details of which were described in our previous study [5]. The chemical composition of the extra-low interstitial Ti-6Al-4V (Grade 23) followed the ASTM F3001 standard specification spherical powder and was supplied by Arcam AB Company, and the powder size distribution was quoted as 45–105 μm. The EBAM process was conducted in a vacuum chamber with a pressure below 5 × 10^−3^ mbar in the beginning, while the pressure at the finishing stage was 2 × 10^−5^ mbar. Each powder layer of 50 μm thickness was created by the raking powder gravity fed from two cassettes, preheated to 730 °C by fast scanning with a defocused electron beam, and the standard parameters for obtaining the porous structure were a beam current at 3 mA and scan speed of 1500 mm/s with a fixed beam diameter of 100 μm and hatch spacing of 150 μm, thereby melting the selected layer areas driven by the three-dimensional CAD model. The preheating and melting processes were achieved by the energy transfer from a high-energy electron beam onto the powder bed. The recycling of the nonmelted powder and/or sintered powder was achieved via a powder recovery system and a mechanical vibrating sieve with a mesh size of ≤150 μm.

### 2.3. Compressive Stress–Strain Testing

The static properties of the porous structures were measured using a static test machine (Shimadzu UH, 1000 kN load cell) by applying a constant deformation rate of 1.0 mm/min. Three specimens were tested for each variation in the porous structure. Uniaxial engineering compression stress–strain curves were obtained, and the mean and standard deviation of each of the five compressive properties were determined. During compressive testing, a digital camera was used to obtain in situ images. Scanning electron microscopy (SEM) secondary electron imaging was carried out on a JEOL JSM-6380 (Tokyo, Japan) at 15 kV for fracture surface observations.

### 2.4. Stress Distribution Simulation

**Figure 2:**
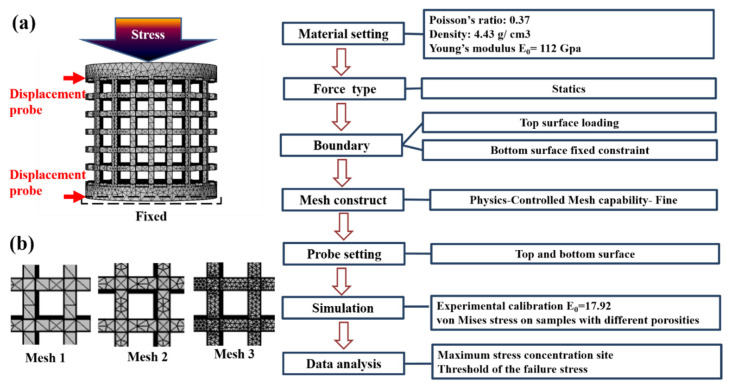
(**a**) Flowchart, meshing and boundary condition of the COMSOL FEA simulation. (**b**) Mesh distribution of different settings: mesh 1 (default, fine), mesh 2 (default, extra fine), and mesh 3 (self-set).

Commercial FEA software COMSOL Multiphysics was adopted to perform the FEA. Instead of a complex computational model, this study uses an elastic model to simulate the collapse behavior of the porous specimens. The experimental flow chart is shown in Figure 2.

Our simulation model was based on the following assumptions:

The deformation of material obeys linear elastic fracture mechanics.
There were no initial defects inside the specimen, and material properties were uniform and isotropic [26].

On the basis of these assumptions, the elastic deformation was assumed to follow Hooke’s law: σ = C·ε, where C is the material’s stiffness matrix, which relates to the stresses, σ, and strains, ε.

The boundary conditions used for the model are shown in Figure 2: (1) the bottom surface was set as fixed constraint; (2) the compressive force was uniformly applied on the top surface of specimens; (3) all nodes and elements above the bottom surface were set as “free”.

To understand the stress distribution on specimens of different porosities, the applied static stresses of the specimens were set up on the basis of their yield strengths: 201, 166, 112, 80, 53, and 43 MPa for C1 to C6, respectively. The material setting of the simulation component was Ti6Al4V grade 23 with a Young’s modulus E_0_ of 112 GPa and a Poisson’s ratio of 0.37.

All CAD models were built by SOLIDWORKS, which accurately matched the actual configuration used in the EBM specimen setup. To confirm if the simulation settings could reach convergence status, three different mesh conditions were used for the C6 specimen with the minimum strut size designed to be 0.6 mm. The detailed mash information and images are shown in Table 2 and Figure 2b. Mesh 1 (physics-controlled mesh capability: fine) set the maximum element size of 2.4 mm and the minimum size of 0.3 mm, which is twofold larger than the minimum strut size of the C6 specimen. Under this setting, 47,565 tetrahedron meshes and 15,065 nodes were established. Mesh 2 (physics-controlled mesh capability: extra fine) set the maximum element size of 1.15 mm and the minimum size of 0.045 mm, which resulted in 151,909 tetrahedron meshes and 41,547 nodes. Mesh 3 set the maximum element size of 0.5 mm and the minimum size of 0.2 mm, which resulted in 1,341,348 tetrahedron meshes and 277,728 nodes. There were four integration points between two nodes for all conditions, and their stress calculation results are also shown in Table 2. The simulation difference for different mesh conditions was less than 3%, implying that the meshing condition of the mesh 1 was sufficient for this study; thus, the C2–C5 specimens used this as the mesh setting.

Moreover, to verify and confirm the strain of a component, two probes were placed on the top and bottom surfaces to obtain the position information. The specific Young’s moduli (E_0_) of the porous specimens were obtained from the set stress/probe detected strain and compared with the experimental results for the purpose of calibration. In this study, the calibrated E_0_ (17.32 GPa) was used to calculate the von Mises stress, including the stress contour and stress histogram. The stress contour (distribution) figures were directly calculated using COMSOL, and the raw data were expressed in the stress histograms.

## 3. Results and Discussion

### 3.1. Compression Stress–Strain Curves

**Figure 3:**
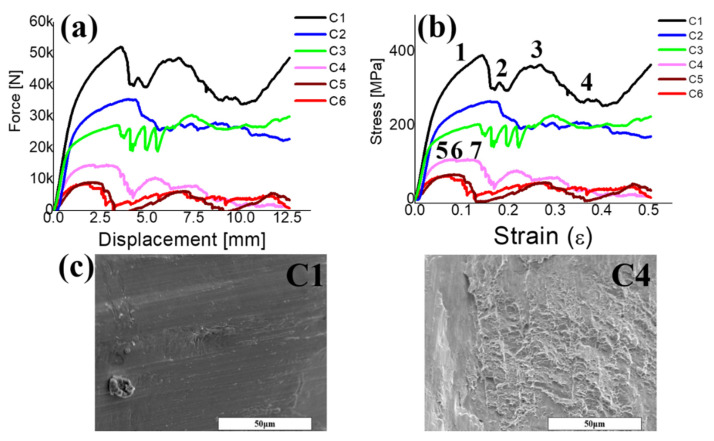
Compressive curves for Ti6Al4V porous specimens with different porosities fabricated by EBAM. The porosities of specimens: C1—25%, C2—33%, C3—40%, C4—55%, C5—59%, and C6—63% in (**a**) force vs. displacement and (**b**) engineering stress vs. engineering strain. Before and after elastic deformation with post collapse denoted, respectively, in (1)–(4) and (5)–(7) strain stress curves in the C1 and C4 specimens. Fracture surface in (**c**) shows the smooth surface of C1 and the ductile dimples of C6 specimen.

Compression tests were conducted on six specimens with different porosities. The load–displacement and stress–strain curves of all specimens shown in Figure 3a,b were found to start with an elastic zone, where their stress increased linearly up to the first peak and then decreased, followed by a plateau zone with the strain ranging from 0.2 to 0.4, and then followed by multiple peaks in which the stress fluctuated, ending with a densification zone, where stress increased linearly. The fracture morphology was observed in the SEM images shown in Figure 3c. Ductile dimples and a smooth surface were found at the fracture surface for the C6 and C1 specimens, showing a decrease in the porosities. For C1, the fluctuation became large in the plateau zone, indicating that the specimens became more brittle. This phenomenon was consistent with the observations of Cheng et al. [27], in which a stochastic foam and reticulated mesh porous structures were fabricated by EBAM, and Choy et al. [28], where cubic and honeycomb porous structures were fabricated by SLM.

### 3.2. Compressive Deformation Behavior

**Figure 4:**
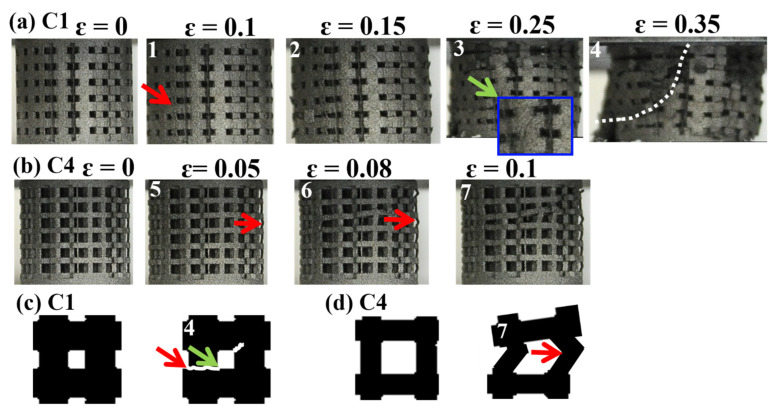
Images of (1)–(4) and (5)–(7) sites in the stress–strain curve in Figure 3. The possible stress concentration region observed here is denoted by red arrows, and the diagonal crack is denoted by green arrows. (**a**) C1 specimen in the elastic region with increased compressive strain: the crack was found at the edge at point 1 (ɛ = 0.1). Struts were over the elastic region where vertical and horizontal struts collapsed at point 2 (ɛ = 0.15) and a diagonal crack at point 3, and the following shear diagonal collapse manner is denoted by the white line at point 4 (ɛ = 0.35). (**b**) C4 specimen with an increased compressive strain: the initial crack is located in the center of the vertical struts at point 5 (ɛ = 0.1); bulking vertical strut deformation then induced horizontal strut collapse at points 6 and 7, leading to layer-by-layer collapse. Proposed fracture behavior for C1 specimen in (**c**) and for C4 specimen in (**d**).

The compressive deformation behavior of the porous specimens was recorded using a high-resolution digital single-lens reflex camera. Two types of deformation were found in the visual observation during the compression test, as shown in Figure 4. The specimens with low porosities, C1, C2, and C3, exhibited abrupt shear failure with one diagonal shear band forming throughout the entire specimen. For the typical low-porosity C1 specimen in Figure 4a, the first collapsed layer started from point 1 of the layer at the edge or adjacent to the edge of the specimens. The next layer to collapse was the adjacent layer, and subsequent collapse of the layers either followed a sequential pattern or occurred randomly at other layers in the strain range ɛ = 0 to 0.2 (point 2). When the strain was increased to 0.3 at point 3, cracks were initiated slightly toward the diagonal direction and diagonal shear band throughout the whole specimen at point 4. In contrast, the high-porosity specimens, C4, C5, and C6, deformed uniformly in a sequential layer-by-layer collapse manner. For the typical high-porosity C4 specimen in Figure 4b, it started with a deformation in the middle part of the vertical struts at point 5, followed by the collapse of all other layers at points 6 and 7. As a result, when the low-porosity specimens deformed as per the shear diagonal model, the stress–strain curves tended to have one large peak, followed by densification with some small fluctuations. This collapse behavior is in good agreement with that observed in porous Ti6Al4 studies [17,28,29]. Combining the strain–stress curve and compressive images, the schematic diagrams in Figure 4c,d illustrate the failure behavior in the low- and high-porosity specimens, respectively. For C1, in the initial period of the linear elasticity and nonlinear behavior, the edges of the V-struts and H-struts began to bend under the compressive loading. After slight bending, some of the struts experienced brittle fractures, resulting in the formation of a diagonal shear band throughout the specimen. For C4, the initial deformation started from the middle of the V-struts, resulting in a layer-by-layer collapse. To further understand the stress behavior of the specimens, stress simulation results are discussed in the next section.

### 3.3. Local Stress Concentration Region Analyzed by FEA

**Figure 5:**
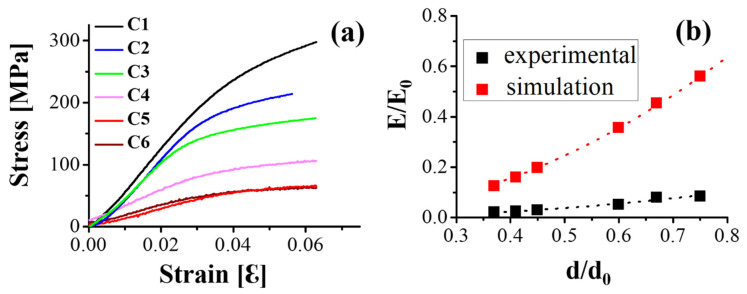
Relationship between the elastic modulus and porosity. (**a**) Compressive stress–strain curve of extracted elastic parts from Figure 3a for the calibration of FEA results; (**b**) comparison between experimental data and FEA results for C1 to C6 based on the Gibson–Ashby model.

The experimental and FEA results of the relative Young’s modulus (E_0_ = 112 GPa) depend on the relative densities of all specimens, as shown in Figure 5. According to the Gibson–Ashby equation [12], their relationship can be described by E/E_0_ = C(d/d_0_)n, where E is the Young’s modulus for a specific density d, d_0_ is the density for a solid specimen, and C and n are constants for a specific structure. After fitting, the n values obtained were 2.02 and 2.08, respectively, for the FEA and experiment results, with the *R*-square values of 0.99 and 0.96, respectively. These results agree with the bending-dominated mechanism of the open cell (*n* = 2) [30]. However, the C values of the FEA and experimental results were approximately 1 and 0.16, respectively, implying that the E_0_ of the FEA should be smaller than that of the ideal Ti6Al4V specimen. For the difference in elastic modulus between the FEA and experiment, studies have reported that cell topologies, strut diameters, cell sizes, bulk geometries, strut defects, porosities, and phase transformations affect the mechanical properties owing to local stress concentrations [20,21,22,23]. To bring the FEA results closer to the experimental results of the real specimens, further simulation used the adjustment of E_0_ as 112 × 0.16 = 17.92 GPa. The calibrated FEA results in Table 3 presenting the elastic modulus for C1 to C6 indicate that the error value of the calibrated E_2_ was less than 13% compared to the experimental value of E.

**Figure 6:**
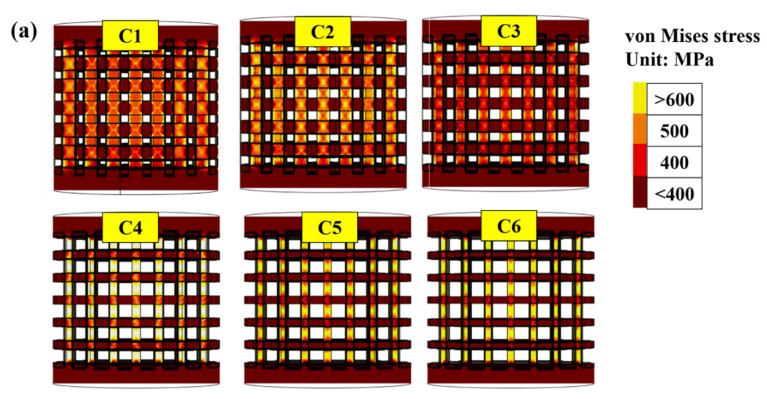

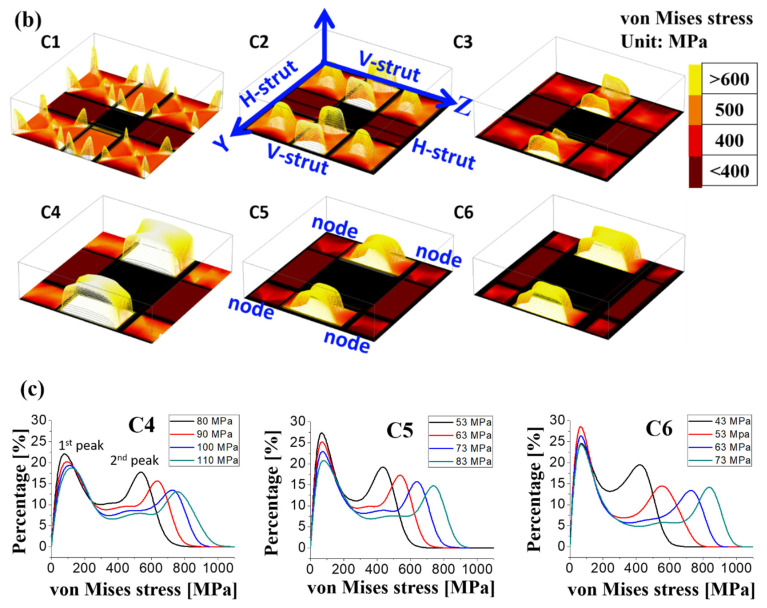
Von Mises stress analysis of the specimens with different porosities using COMSOL software. Side view of the stress distribution for (**a**) whole specimens and (**b**) cells; most stress was concentrated on the vertical struts in all the specimens. X-shaped stress concentration regions were found in C1 and C2 specimens. Stress concentration moved to the center of the struts in C4–C6 specimens. C3 specimen demonstrated a porosity transition. (**c**) Statistical histogram of stress values for the C4–C6 specimens derived from the FEA results. First peak and second peak respectively denote the top surface and strut of the compressive specimen. Applied stress was set on the basis of the yield strength from the experimental values with an increase of 10 MPa for each level.

Figure 6a shows the side view of the von Mises [31] stress distribution (stress counter) for specimens with different porosities. Local stress concentration sites were found at the edge or vertical strut adjacent to the edge of the horizontal strut in specimens C1 and C2. A transition stress distribution was observed in C3 specimen. However, the porosity increased by over 55% (i.e., C4, C5, and C6), with the stress concentration at the center of the strut. The x-shaped stress distribution behavior in C1 and C2 differed from the stress concentration at the center of the struts in the C4–C6 specimens. To observe the stress concentration site clearly, Figure 6b shows the 3D stress counter of the unit cells for different specimens. The maximum stress value was found on the nodes in C1 and C2, which differed from the site at the strut center in the C4, C5, and C6 specimens, implying that the initial crack site was dependent on the porosity for stress behavior. For C1, the initial crack site and x-shaped stress concentration were as shown in Figure 6a,b, and the initial crack sites started with diagonal cracks throughout the entire specimen, which is consistent with our experimental results in Figure 4a. Conversely, for the C6 specimen, the initial crack site was found at the center of the strut according to the FEA results in Figure 6; the sequence of the layer-by-layer collapse was also consistent with the experimental results in Figure 4b.

Sun et al. [32] studied the mechanical properties of porous materials fabricated by additive manufacturing; the stress–strain curve and the following fracture morphology showed brittleness with nonobvious plastic deformation, implying that the yield strength was nearly at the ultimate value. As shown in Figure 3 and Figure 4, all the specimens showed brittle failure without plastic deformation, especially in the high-porosity C4–C6 specimens. Therefore, the yield strength was adopted to obtain the stress concentration values to predict the threshold values of the failure. Figure 6c shows the statistical histogram of the stress values for C4, C5, and C6, based on the FEA results. The first loading stress of the specimens depended on their yield strengths of 80, 53, and 43 MPa for C4, C5, and C6, respectively. All the specimens showed a typical double peak distribution, representing the top surface (first peak) and the V-strut center (second peak) of the compressive specimens. The V-strut center withstood the stress of 400–600 MPa at the first level of applied stress, which was much smaller than the compressive yield strength of solid Ti6Al4V grade 23 at 860 MPa [33]. Owing to the brittleness, the failure strength was assumed as 800–900 MPa, and the applied stress was based on the yield strength at four levels. Each level was increased by 10 MPa, and the applied stress causing the local stress over the failure strength was called the threshold stress. As the applied stress increased from levels 1 to 4 in each specimen, the stress on the V-strut also increased. The threshold applied stresses were 100, 83, and 63 MPa for C4, C5, and C6, respectively, which were close to the experimental results (110, 68, and 62 MPa, respectively).

**Figure 7:**
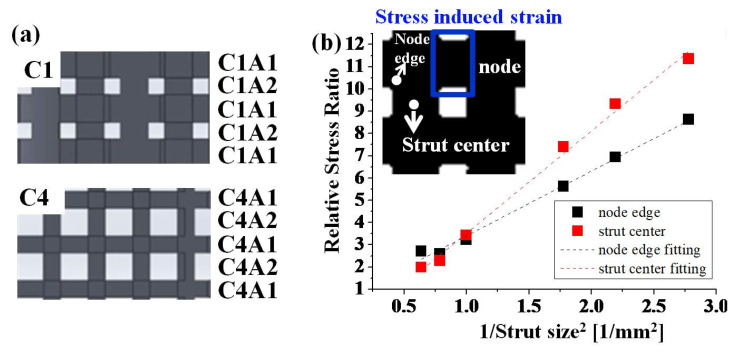
Proposed mechanism for stress concentration site shift. (**a**) Cross-sectional areas of H-strut and V-strut were named C1A1, C1A2 in C1 and C4A1, C4A2 in C4. (**b**) Relationship between the relative stress and sites of nodes and struts in all specimens. Junctions of the struts are named nodes. Relative stress ratio is the ratio between the yield strength for a particular porosity specimen and the stress of the particular site.

The stress-concentrated site shifting phenomenon can be explained by the geometry of these specimens. As shown in Figure 7a, these porous specimens were recognized as a series of materials with H-strut and V-strut cross-sectional areas (A1, A2). The cross-sectional area A2 of the V-strut was low, approximately 1/2 A1 for the C1 specimen. Therefore, the stress on A2 was twice that on A1. However, node A1 showed an additional stress on the edge owing to the shear-induced strain area, and this stress concentration phenomenon (k factor) has been widely studied [34]. The stress values at the strut center and node edge were obtained from Figure 6b to discuss the stress on the strut size. To simplify, the stress obtained above should be divided by the yield stress 201, 166, 112, 80, 53, and 43 MPa for C1 to C6, respectively, to obtain a relative stress ratio before comparison. Figure 7b shows the 1/strut size^2^ values, depending on the relative stress ratios of different sites. Both sites showed a linear relationship with the 1/strut size^2^, although the slopes differed. The stress values should be related to the strut areas, and the obtained FEA result is quite reasonable. The stress on the strut center was smaller than that at the node edge at the beginning (C1), but it increased rapidly with the strut size. When the strut size was between 1.125 mm (C2) and 1 mm (C3), the stress on the strut center was larger than that at the node edge. This is the reason for the changes in the stress concentrations with the changes in porosities.

Using the above two types of deformation in the low- and high-porosity specimens, the stress behavior and initial crack site using the elastic model instead of the plastic model were validated. To the best of our knowledge, a method for predicting the initial crack site of a porous material has not been reported thus far. It is assumed that the elastic model of the FEA for a brittle material after the EBAM process without heat treatment is used to calculate the stress behavior and predict the initial crack site. Compared to previous research reporting on the plastic model of FEA to predict mechanical properties [20,21,22,23], initial stiffnesses, and plastic collapse strengths [24], as well as micro stress analysis using a multiscale simulator with micro-CT, which provided an FEA to predict the strength [35], the method demonstrated in this study presents a simple way to predict the initial crack site of a porous structure. Such numerical results would contribute to the design of porosities and Young’s modulus for the studying of fracture mechanisms in future.

## 4. Conclusions

In this study, we coupled compression curves and images with FEA to investigate the failure mechanisms and initial crack sites of Ti6Al4V porous structures with cubic units, whose porosity ranged from 25% to 63%, fabricated by electron beam additive manufacturing. From this study, we reached the following conclusions:

1. The deformation at low porosities showed a shear diagonal pattern, and the structure was brittle with a high fluctuation peak in the plateau of the compressive stress–strain curve. With an increase in porosity, the deformation behavior shifted toward a layer-by-layer collapse and showed a uniform fluctuation in the plateau region.
2. In terms of the elastic model (FEA), in the low-porosity specimens, where the stress statics diagram showed a normal distribution, most of the stresses were smaller than the yield strengths. The maximum stress concentration was located at the edge of the strut and the node. In contrast, in the high-porosity specimens, the stress statics shifted the low and high stresses into two peak distributions, and the maximum stress concentration was located at the center of the struts.
3. The applied threshold stresses were 100, 83, and 63 MPa for C4, C5, and C6, respectively, which were close to the experimental results (110, 68, and 62 MPa).
4. By combining the compressive experimental results and FEA results, the initial failure sites of the low-porosity specimens were demonstrated to be located at the edge of the struts in the FEA results; the structure collapsed diagonally with the formation of one shear band throughout the specimen. However, the high-porosity specimens possessed maximum stresses at the center of the struts; the structures collapsed layer-by-layer.

## Figures and Tables

**Table 1:** Structure identification and dimensions with a fixed unit cell of 4 mm of the specimens tested. D: pore diameter. S: strut diameter. DPW: designed porosity of the specimen.

	D (mm)	S (mm)	DPW (%)
C1	1.50	1.25	25
C2	1.75	1.13	33
C3	2.00	1.00	40
C4	2.50	0.75	55
C5	2.65	0.68	59
C6	2.80	0.60	63

**Table 2:** Mesh information and calculation stress of the different mesh conditions.

Mesh Condition	Element Size (mm)		Mesh Results (Count)		Calculation Stress (MPa)	
	Maxima	Minimum	Tetrahedron	Node	Strut Center	Node
Mesh 1 (default, fine)	2.4	0.3	47,565	15,065	488	371
Mesh 2 (default, extra fine)	1.15	0.45	151,909	41,547	484	383
Mesh 3 (self-set)	0.5	0.2	1,341,348	277,728	488	378

**Table 3:** Calibrated Young’s modulus E_2_ of the FEA using the corrected E_0_ = 17.92 GPa compared to the Young’s moduli obtained experimentally and the non-calibrated E_1_ using the original E_0_ = 112 GPa.

Specimen	C1	C2	C3	C4	C5	C6
E—Experimental (GPa)	9.46 ± 1.08	8.93 ± 0.88	5.67± 0.39	3.29 ± 0.46	2.73 ± 0.25	2.39 ± 0.33
E1—FEA (GPa)	62.79	50.81	39.91	22.17	17.9	13.97
E2—FEA (GPa)	10.05	8.13	6.39	3.55	2.86	2.24
